# Consumer store experience through virtual reality: its effect on emotional states and perceived store attractiveness

**DOI:** 10.1186/s40691-021-00256-7

**Published:** 2021-05-05

**Authors:** Byoungho Jin, Gwia Kim, Marguerite Moore, Lori Rothenberg

**Affiliations:** 1 Albert Myers Distinguished Professor, Department of Textile and Apparel, Technology and Management, North Carolina State University, Raleigh, USA; 2 Doctoral Candidate, Department of Textile and Apparel, Technology and Management, North Carolina State University, Raleigh, USA; 3 Professor, Department of Textile and Apparel, Technology and Management, North Carolina State University, Raleigh, USA; 4 Associate Professor, Department of Textile and Apparel, Technology and Management, North Carolina State University, Raleigh, USA

**Keywords:** Virtual reality, Store attractiveness, Emotions, Sentiment analysis, Semantic network analysis

## Abstract

Based on the stimuli-organism-response model, this study aims to examine whether consumers’ store experience through virtual reality (VR), compared to website experience, can attract them enough to perceive the online store as appealing. Two types of stimuli were developed for the experiments: consumers’ VR store experience (106 data) (i.e., having respondents experience 360-degree-based VR store videos recorded at a fashion retailer) and store website experience (107 data) (i.e., having respondents experience the same store’s website). The results revealed that relative to an ordinary store website, consumers’ VR store experience evoked positive emotions and increased perceived store attractiveness. This study also discovered that store familiarity does not moderate the relationship between the two store experience types and evoked emotions, implying that VR technology is effective regardless of consumers’ familiarity with a store. Text analytics were also utilized, providing additional insights about their VR store experiences. This study suggests an effective method for online retailers to emulate an attractive store environment and entice consumers through VR, regardless of the retailers’ fame. Specifically, it demonstrates the effectiveness of VR over website in enhancing store attractiveness, an under-studied area.

## Introduction

Retailers invest in creating an attractive store environment because it induces consumers to visit and provides them with an enjoyable shopping experience (e.g., Baek et al., 2018; Darden et al., 1983; Orth & Wirtz, 2014). Retailers, however, cannot fully portray their store environment through their websites. One way to offer a unique store experience online is using virtual reality (VR). By replicating the real world (Herz & Rauschnabel, 2019; Steuer, 1992), VR enables consumers to experience the store atmosphere without actually visiting the store. A growing number of retailers are testing VR online. One example is eBay, which collaborated with Myer, an Australian department store, and created a VR environment on eBay’s website to allow its consumers to explore the store environment virtually (Team, 2016). As with this case, VR can be an effective tool in delivering store atmosphere and providing unique shopping experiences.

There are several types of VR. Among them, a viable way for any retailer to utilize easily is the 360-degree-based VR videos because these videos require fewer skill sets and financial budgets to operate than other VR types. To create 360-degree-based VR videos, retailers can use 360-degree cameras that can be used to record in-store environments. The cameras are inexpensive and easy to use. To play VR videos, consumers can use any VR headsets, even mobile-based VR operators, such as Google Cardboard (Jang et al., 2019). These VR devices are also easy to use. By playing VR videos, consumers can experience the store virtually and overcome the physical limitation of geographical locations. Therefore, this study posits that this type of VR is practical for retailers.

With the emergence of VR and its use in practice, several academic studies have emerged on the use of VR in retailing and marketing. Most of these studies have paid attention to VR-led consumer responses, such as behavioral intention and customer satisfaction (Domina et al., 2012; Gabisch, 2011; Lau & Lee, 2018; Pizzi et al., 2019), but do not fully explain why the VR store environment results in positive behavioral intentions. Another research gap is that previous VR studies have not considered the potential impact of consumers’ level of familiarity with the store in question. Positive emotions, such as excitement, arousal, and pleasure, may depend on consumers’ prior exposure to the store. That is, consumers’ prior experience with the store may lessen the novelty of experiencing the store a second or subsequent time through VR. To evaluate the effect of VR store experience accurately, this study employs consumers’ store familiarity as a moderator. The third research gap pertains to the way the VR store environment is created in previous studies. Most of the earlier studies created a VR store environment digitally with graphic tools (e.g., Pizzi et al., 2019; Van Herpen et al., 2016) or web platform (e.g., Domina et al., 2012; Gabisch, 2011), rather than creating it in a real retail store (Domina et al., 2012; Gabisch, 2011; Lau & Lee, 2018; Pizzi et al., 2019; Van Herpen et al., 2016). This approach may have a limitation in representing a physical store and thus capturing customers’ positive emotions because the store environment is less authentic. Also, this approach requires more budgets and skill sets for retailers to operate. Related to this, previous VR studies call for research in a more reality-oriented store setting (Lau & Lee, 2018).

Addressing the research gaps, this study builds a conceptual framework based on the stimuli-organism-response (SOR) model (Mehrabian & Russell, 1974) and schema theory and tests it through experiments. The framework specifies whether VR can deliver a physical store atmosphere better than a website to increase the consumers’ emotional states, which in turn helps consumers perceive the store as attractive. The SOR model explains that the stimuli (i.e., VR versus website store experience) affect organisms (i.e., emotional states), and the organism influences consumer responses (i.e., store attractiveness). Schema theory is employed to explain whether consumers’ elicited emotional states (i.e., arousal and pleasure) vary with their familiarity with the store (Kent & Allen, 1994). In addition, this study aims to understand more about consumers’ VR store experience through text analytics.

This study contributes to the literature by observing how consumers’ emotional states lead to perceive a boutique store attractiveness, which has been less studied in the literature. Comparing the 360-degree-based video driven VR store environment created in an actual store, (rather than a digitally created store), with website experience with the same store will extend the literature by discovering the effectiveness of VR over websites in conveying the store atmosphere. Further, the comparison of VR with website store experiences will suggest whether VR store experiences can overcome the limitation of websites and lead consumers to perceive greater store attractiveness. The findings of this study further suggest whether the volume of benefits driven by VR store experiences vary with customers’ store familiarity level, by including it as a moderator.

This study is tested in a small independent fashion boutique that maintains both its own brick-and-mortar store and website. An additional reason for the selection of a small fashion store is that consumers’ familiarity with the store may vary greatly, compared to a large retail store mostly known to many people.

## Literature review

### Theoretical framework

#### Stimuli-organism-response (SOR) model

This study is built on the stimulus-organism-response model (SOR) by Mehrabian and Russell (1974). According to the SOR model, the environment that contains stimuli (S) can influence people’s organisms (i.e., internal states) (O), which subsequently causes people’s responses (R). Specifically, stimulus is referred to as the factor that influences an individual’s internal states, which means an organism. Organism represents individual emotional reactions to the environment (Mehrabian & Russell, 1974). When an individual encounters a stimulus (S), he or she can form or change his or her internal states (O). Here, response (R) is the individual’s reactions driven by the organism. Researchers further identified three dimensions of affective responses (i.e., organism) triggered by stimuli; pleasure, arousal, and dominance (i.e., PAD) (Mehrabian & Russell, 1974). Pleasure and arousal are found to be two important variables that explain consumer behaviors (Russell & Pratt, 1980); we hence focus on these two affective responses. Arousal is an affective state “ranging from sleep to frantic excitement” (Mehrabian & Russell, 1974, p. 18). It describes a person feeling simulated or alert when exposed to a stimulus. Pleasure is a person’s hedonic state—feeling happy and joyful when exposed to a stimulus (Mehrabian & Russell, 1974).

In retail and marketing research, the SOR model has been actively utilized to explain the impact of the store environment on shopping behavior (Vieira, 2013). Elements of the store environment, including color, temperature, spatial and temporal components, are considered as stimuli in many previous studies (Vieira, 2013). The SOR literature also provides research insight into atmospheric stimuli such as music and scent (Roschk et al., 2017) and advertising stimuli (Min et al., 2019). Arousal and pleasure represent the most commonly evoked emotions, also some studies focus on different primary emotional responses such as dominance (Vieira, 2013), satisfaction (Roschk et al., 2017), and attitudes (Min et al., 2019). In the retailing context, the responses evoked by emotional states incorporated behavioral intentions (Roschk et al., 2017), buying intention (Min et al., 2019), flow (Wang et al., 2007), and shopping motivation and value (Babin et al., 1994; Wang et al., 2007).

In earlier studies, stimuli meant external marketing mix variables or other environmental inputs that can affect consumers (Bagozzi, 1986). As with previous studies that considered store environments as stimuli (e.g., Donovan & Rossiter, 1982, 1994; Huang & Liu, 2014; Watson et al., 2018), stimuli in this study consist of respondents’ store experiences through either VR or website. The organism is ‘arousal’ and ‘pleasure’, following the previous studies (Mehrabian & Russell, 1974) and response is ‘store attractiveness’ that can be evoked by the consumers’ emotional states.

#### Schema theory

While the SOR model builds the overall research framework, schema theory (Dahlén et al., 2005; Kent & Allen, 1994) is applied to describe the moderation effect of store familiarity in consumers’ emotional states on store experiences. The concept of schema was first introduced by Bartlett (1932), and many researchers developed and elaborated it (Kent & Allen, 1994; Piaget, 1953; Rumelhart, 1980). Schema is an active framework created by past experiences and reactions in one’s procedural memory that can help to understand a new experience with the expectations shaped by the framework (Bartlett, 1932). Schema was then considered as a key concept of cognitive science (Piaget, 1953; Rumelhart, 1980). According to Rumelhart (1980), the schema theory explains how the brain organizes knowledge. All knowledge is packed into units, which become schema, which is stored in memory to be a data structure representing knowledge about all concepts. In other words, underlying objects, situations, events, sequences of events, actions, and sequences of actions are stored in memory, and these saved underlying experiences help to understand new experiences. Thus, schema construe a network of associations that serve as a guide for a person’s perception (Bem, 1981). Incoming stimuli are processed in an individual’s perception based on the individual’s existing schema (Bem, 1981).

The schema theory has been used for exploring consumers’ product or brand familiarity, as a consumer tends to form a set of expectations about a product category through previous experiences, and these expectations develop the perceptions of product and brand attributes (Sujan & Bettman, 1989). Consumers utilize schemas to recognize, understand, and judge a new product or a brand because they can recall previous associations already stored in their memory (Heckler & Childers, 1992; Lange & Dahlén, 2003; Low & Lamb, 2000; Sujan & Bettman, 1989). Further, when new information that is not consistent with the present brands and conflicts with previous schemas is provided, consumers can choose to either assimilate or accommodate, so that it can be incorporated into the current schema or accommodate the new information to modify the present schema, in order to understand the new information (Sujan & Bettman, 1989). In the current research, the schema theory explains why less familiar stores can achieve more benefits by using VR technology than famous retailers. The level of store familiarity is included as a moderator to observe whether the effectiveness of VR store experience in evoking consumers’ emotional states can be strengthened if the store is unfamiliar and new to the consumers.

### Types of virtual reality

As per previous studies, VR can be categorized into immersive VR and non-immersive VR (Mills & Noyes, 1999; Park et al., 2018). This classification is based on how realistically the VR type can respond to VR users’ movements, and the virtual environment’s scale (Mills & Noyes, 1999; Park et al., 2018). Immersive VR can operate more realistic VR experiences because the virtual world completely surrounds the VR users (Mills & Noyes, 1999; Park et al., 2018), while non-immersive VR allows VR users to experience virtual spaces through a flat screen (i.e., a monitor) (Costello, 1997). Therefore, immersive VR requires high-performance software and equipment, such as head-mounted displays (HMDs) to surround users, but non-immersive VR does not need them (Costello, 1997). In immersive VR, HMDs can sense VR users’ movement and alter the views and orientation of VR environments to address the movements (Meissner et al. 2017), so it has a higher influence on VR users than non-immersive VR (Alshaer et al., 2017; Park et al., 2018).

Another way VR in retailing and marketing is classified into two levels based on the product involvement system (Cowan & Ketron, 2019). When VR users are allowed to engage more in observing each individual product shown in the virtual world, it has a high level of involvement. On the contrary, in low involvement VR type, VR users cannot access each product presented in the virtual world.

This study uses 360-degree-based VR videos played through HMDs. VR users of this type are fully surrounded by virtual environments using HMD, so it is categorized as immersive VR. At the same time, VR users cannot select, touch, or manipulate each product in this VR type, so it is classified as a low-involvement VR.

### Use of virtual reality in practice

Recently, VR has been noted for its use in marketing and retailing to offer richer consumer experiences (CB Insights, 2018a; Cognizant Reports, 2016), among apparel and fashion retailers. VR users can virtually explore and shop as they access stores using headsets without physical visits (CB Insights, 2018b; Grieder et al., 2014). The tourism industry is one of the fields actively using VR. For example, Marriott Hotels partnered with Samsung to launch VRoom Service, which enables Marriott guests to request delivery of a VR headset to their room using the hotel’s mobile app, through which they can virtually explore destinations. The pre-filmed VR videos of travel destinations are saved in the VR headset so that guests can easily immerse themselves in the virtual tours. Compared to the limited experience conveyed through static photos, the VR experience can offer more realistic experiences to guests (Stephens, 2017).

More recently, Amazon opened its VR kiosks in 10 shopping malls to promote the Prime Day shopping event (Horwitz, 2018). Customers wearing VR headsets walked through virtually created rooms reflecting Amazon store sections, such as fashion, bath and beauty, and toys. These customers used VR controllers to handle any product in full three-dimensional view.

Apparel and fashion retailers also use VR technology to enlarge consumers’ shopping experience. TopShop and Rebecca Minkoff offered VR fashion show to their customers, affording the customers a 360-degree view of the products (Milnes, 2017). Rebecca Minkoff further attempted to introduce this advanced technology to commerce, so that consumers can enter the VR world and shop as if they are in a real shopping setting (Milnes, 2017). A shoe brand, TOMS brought VR to provide trips to retail customers (Nafarrete, 2015). In its flagship store in Los Angeles, consumers could watch a four-minute 360-degree film (i.e., VR video) that allowed them to virtually travel along with TOMS in Peru. TOMS’s policy is to donate a matched pair of shoes to a child whenever a pair of shoes are sold to a customer, and the VR video shows how their donation helps Peru.

Online retailers have also accepted the technology. eBay, an online retailer, partnered with Myer, an Australian fashion retailer, to launch a VR departmental store (Team, 2016). Australian consumers can get an immersive shopping experience of a Myer’s VR store, as they browse and purchase Myer’s items online. Myer’s VR store, based on the eBay platform, enables consumers to view items just as though they were in the physical store. Similarly, Alibaba’s Taobao consumers are able to walk through the virtual online mall with a 360-degree panoramic view (Zuo, 2016). Despite the active use of VR in larger companies, smaller retailers have rarely adopted the technology yet.

As VR can offer immersive and interactive experiences, companies tend to apply the technology to provide virtual tours and enhance the online shopping experience (Cammareri, 2019; McDowell, 2020). Companies frequently partner with an independent VR platform provider to implement these activities. For example, the Tommy Hilfiger brand, partnered with Obsess, a VR platform enabler, to create a successful implementation of virtual tours. Allison Mitchell, a handbag designer brand, also partnered with Obsess to create a 360-degree virtual storefront (Mihalic, 2019). Furthermore, as VR requires minimal physical interaction, its use is expected to increase in retailing during the COVID-19 pandemic (Rueter, 2020; Sandel, 2020).

### Academic studies on VR in retailing and marketing

Earlier studies on VR are summarized in Table 1. As can be seen from the Table, VR-related studies focused on shopping settings in various product categories: apparel (e.g., Domina et al., 2012), food (e.g., Pizzi et al., 2019) and IT (Gabisch, 2011). These previous studies have conducted experiments mainly to observe how the VR experience can be effective in retailing and marketing, mostly relying on digitally-created virtual stores using graphics to mimic actual shopping places (Lau & Lee, 2018; Pizzi et al., 2019; Van Herpen et al., 2016). For example, Van Herpen et al. (2016) built a store interior and store display using 3D graphic design, to generate food products and layout of the products, mirroring an actual grocery store. Some researchers utilized virtually-created retail store at Second Life, which is a platform that allows brands to create their own virtual world using an avatar and 3D graphically-created places (Domina et al., 2012; Gabisch, 2011). For example, Gabisch (2011) asked respondents to visit brand stores (e.g., Adidas and IBM) created virtually at Second Life.

**Table 1:** Experimental studies on the virtual reality experience in shopping context

Study	Context	Stimuli	Focus
Gabisch (2011)	Apparel, Food, IT	3D online virtual world (Second Life) experience	Real world purchase intention, real world purchase behavior
Domina et al. (2012)	Apparel and fashion	3D online virtual world (Second Life) experience	Shopping intention within the 3D online virtual world
van Herpen et al. (2016)	Food products	Comparison among physical-store vs. virtually created VR-based store vs. pictorially represented store shopping experience	Similarity between real-life and VR shopping behavior
Lau and Lee (2018)	Apparel	Virtually created shopping mall experience	Purchase intention, interactive and hedonic shopping experience
Park et al. (2018)	Apparel	VR model store visits using HMD	Purchase intention
Pizzi et al. (2019)	Food products	Comparison between physical-store vs. virtually created VR-based store shopping experience	Store satisfaction
Jang et al. (2019)	Footwear	VR store using mobile-based HMDs	Experiential shopping value, Approach intention
Baek et al. (2020)	Apparel	360-degree VR photos	Brand equity, visit intentions

Developed by authors based on literature review

Earlier studies listed in Table 1 confirmed that VR experience can drive purchase intention (Lau & Lee, 2018; Park et al., 2018) and shopping intention (Domina et al., 2012) in the virtual world. Especially, Domina et al. (2012) discovered that consumers’ heightened enjoyment through VR experience led to higher shopping intentions. Further, Gabisch (2011) found that the virtual store experience resulted in real-world visit intention and purchase behavior. In food shopping context, two studies investigated whether VR store experience is perceived similar to a real, physical store experience through a comparison (Pizzi et al., 2019; Van Herpen et al., 2016) and confirmed that VR store experience could offer customer satisfaction (Pizzi et al., 2019).

While these studies offer a preliminary understanding of the role of VR in shopping contexts, a couple of areas are worthy of research attention. First, earlier VR studies were mostly limited to customers’ behavioral intentions (e.g., purchase intention), rather than the customers’ perceptions of the store atmosphere. VR capabilities that replicate the real world could effectively help consumers experience the store atmosphere, which further facilitates positive evaluations toward the store, such as perceived store attractiveness. Perceived store attractiveness is defined as the consumers’ perception of the extent to which a store is appealing to generate positive evaluation (Baek et al., 2018; Orth & Wirtz, 2014). Consumers’ perceived store attractiveness is an important factor in retailing, as it can lead to store patronage intentions (e.g., Darden et al., 1983), re-patronage intention (e.g., Orth & Wirtz, 2014), and approach-avoidance behavior (e.g., Baek et al., 2018; Orth & Wirtz, 2014). Despite the potential use of VR in creating a store atmosphere and subsequent positive evaluation towards the store, earlier literature has paid little attention to this aspect.

Second, experiencing a store through VR is still a novelty to the majority of consumers (Pizzi et al., 2019). Therefore, just as other technology can provide shoppers with positive emotions (e.g., pleasure) by evoking sensory and affective experience (e.g., Dennis et al., 2014), so can VR produce positive heightened emotions (Park & Im, 2016; Park et al., 2018). Related to this reasoning, consumers’ familiarity with the store may reduce the novelty of the VR store environment, thus impacting the magnitude of consumers’ emotions. Nonetheless, earlier studies listed in Table 1 disregarded the influence of consumers’ store familiarity level.

Third, most VR studies in shopping settings used digitally-created VR graphic stores (Domina et al., 2012; Gabisch, 2011; Lau & Lee, 2018; Pizzi et al., 2019; Van Herpen et al., 2016). The use of VR-view videos showing actual places is limited to the tourism field (Deng et al., 2019; Griffin et al., 2017). In apparel retailing, Baek et al. (2020) conducted a study of virtual tours using 360-degree photos of a physical store. However, though they used VR with an actual store, they did not incorporate video stimuli, which can provide more realistic experiences than static photos. To reduce the gap between actual store experience and virtual store experience, this study posits that it is helpful to use VR video stimuli that record and capture the actual store, rather than a graphically-created one.

Considering all the aforesaid, this study investigates whether consumers’ perceived store attractiveness can be increased by VR-heightened emotions (i.e., arousal and pleasure). Specifically, by comparing consumers’ store experiences through VR that features the physical store interior of a real fashion boutique vs. website of the store, this study examines the role of VR-view store experience in conveying actual store environments to please and arouse consumers, which in turn helps consumers feel the store more attractive. This study further tests whether consumers’ store familiarity level carries some weight relating to VR store experience, which was not considered in earlier literature. In order to better understand consumers’ opinion on VR store experience, this study includes text analytics answered an open-ended question.

### Research framework and hypotheses development

**Fig. 1:**
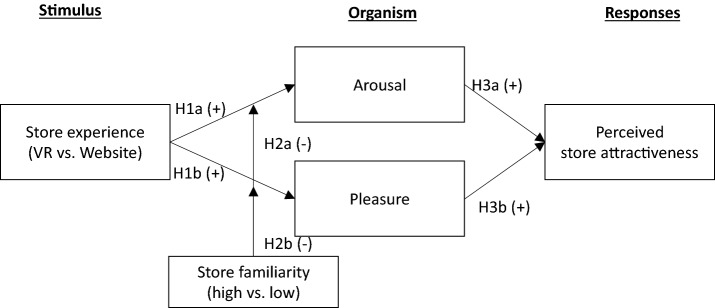
Conceptual framework. *In measuring store experiences, VR store experience is coded as 2, website store experience is coded as 1. *Store familiarity: moderator

Based on the SOR model by Mehrabian and Russell (1974), this study posits that consumers’ store experience (S) (i.e., VR vs. website) triggers an emotional state (O) (i.e., arousal and pleasure), and subsequent response to the store (R) (i.e., perceived store attractiveness). Further, the relationship between store experiences and emotional states is expected to be moderated by store familiarity, applying the schema theory. Figure 1 shows the research framework and hypotheses.

Donovan and Rossiter (1982) and Mehrabian and Russell (1974) proposed that the amount of information in the environment (i.e., information rate) decides the level of consumers’ affective reactions and a newer environment elicits greater emotional states. The information rate is referred to as its degree of novelty and complexity. Novelty includes new and unexpected environmental settings, and complexity means “the number of elements or features” (Donovan & Rossiter, 1982, p. 40).

This study expects that VR store experience offers higher novelty and complexity to consumers and that such experience will provide more detailed information than websites. Specifically, by wearing VR headsets, consumers can have a 360-degree view of the store and virtually experience the store atmosphere, while websites cannot convey the environment of the physical store adequately. In the virtual world, consumers can encounter more “number of elements or features” that replicate the real world. Indeed, the VR shopping experience can overcome the shortcomings of traditional online shopping, such as a lack of realistic view and feeling the presence with a virtual affluence (Van Herpen et al., 2016). Consumers too may not expect that they will directly feel the store atmosphere through virtual media, so the VR experience will be a new and unexpected experience for them.

Taken together, this study posits that because VR offers a higher information rate and new experience than a website, consumers’ VR experience will cause them greater arousal and pleasure than a website experience. Thereby, H1 is developed as follows.

*H1*: VR store experience leads consumers to greater (a) arousal and (b) pleasure than website experience.

Based on the Schema theory, researchers have found that consumers usually form more elaborate and sophisticated schema towards familiar brands, compared to unfamiliar brands (Heckler & Childers, 1992; Kent & Allen, 1994; Low & Lamb, 2000). This is because they possess more associations for a familiar brand (Low & Lamb, 2000), and the knowledge of a brand can be easily retrieved from a familiar brand, rather than a less familiar brand (Heckler & Childers, 1992). Higher brand familiarity could be formed from a stronger schema consisting of many previous experiences related to the brand (Kent & Allen, 1994) so that this strongly formed schema of a familiar brand is difficult to change with a single additional encounter.

On the contrary, consumers’ weaker schema towards less familiar brands can be easily influenced by a single new encounter, such as a new experience (Dahlén et al., 2005; Klein et al., 2016). Consumers form a less sophisticated and less established schema about unfamiliar brands, vis-a-vis the well-known brands (Dahlén et al., 2005; Kent & Allen, 1994). Therefore, a weak schema towards a less famous store can be influenced by a new experience (i.e., VR) easily, compared to the famous stores.

Accordingly, this study posits that consumers may possess altered impressions of the store image and feel higher enjoyment when they virtually experience relatively less-known stores, compared to familiar stores. Following this, when consumers are less familiar with the store, a single store experience can have a greater influence on their responses to the store. Similarly, consumers with weaker store familiarity may feel VR experience to be more novel to them than those with higher store familiarity; thus, the former feels a more heightened emotional state.

*H2*: Store familiarity moderates H1 such that lower store familiarity leads to greater (a) arousal and (b) pleasure.

Store-evoked arousal and pleasure are known to increase positive consumer responses to the store (Ha & Lennon, 2010). Arousal and pleasure evoked in the store or Web environments are positively related to consumers’ desire to explore or shop (Eroglu et al., 2003), satisfaction (Eroglu et al., 2003; Ha & Lennon, 2010), behavioral intention (Ha & Lennon, 2010), and retail choice and retail preferences (Dawson et al., 1990). Arousal and pleasure stimulated by the online store atmosphere, created by background color and music, increase consumers’ behavioral intention and approach (Wu et al., 2008). Pleasure stimulated by shopping experience and technology has a positive effect on consumers’ responses (Das & Varshneya, 2017; Dennis et al., 2014).

Among the consumers’ responses, the study proposes that the arousal and pleasure (Os) enhance consumers’ perceived store attractiveness (R). Perceived store attractiveness is the extent to which a store is appealing to consumers, enough to generate positive store evaluation (Baek et al., 2018; Orth & Wirtz, 2014). Store environments can have an influence on perceived store attractiveness (Baek et al., 2018) and even through emotion, such as pleasure (Orth & Wirtz, 2014). Accordingly, this study expects that once consumers feel arousal and pleasure through experiencing the store atmosphere through VR or a website, they will be more likely to feel the store is attractive.

*H3*: Heightened (a) arousal and (b) pleasure lead consumers to higher perceived store attractiveness.

## Methods

### Overview

A study was designed to discover whether VR store video experiences can evoke consumers’ emotional states, compared to the effectiveness of store website experiences, which in turn increase perceived store attractiveness. For a comparison between VR store videos and store website on emotional states, a one-factor between-subjects experiment was designed. Two hundred and thirteen usable observations (106 from VR and 107 from website) out of 237 items of data were collected from students of a Southeastern University using a convenience sampling. The student sample was selected based on their great interest in or heavy use of technology (e.g., Lee et al. 2006). Data were collected from May to September 2019. Demographic information is summarized in Table 2.

**Table 2:** Descriptive analysis of demographic information (n = 213)

Variable	Category	N	%	Variable	Category	N	%
Age	Mean = 20.94 SD = 2.60 Min. = 18 Max. = 34 N = 213			Ethnicity	Caucasian	143	67.14
					Black or African American	12	5.63
					American Indian or Alaska Native	0	0.00
					Asian	47	22.07
Gender	Female	166	77.93		Native Hawaiian or Pacific Islander	0	0.00
	Male	47	22.07		Other	11	5.16

### Stimuli and procedures

This study tested the VR store experience against the website store experience by conducting experiments using stimuli rather than through measurement by scales. To compare VR and website store experiences relating to a real fashion boutique, two types of stimuli were employed. The VR experience was measured by having consumers experience the VR store video stimuli developed by the authors from a local fashion boutique. The website experience was gauged by having the respondents browse and explore the same boutique’s website, which conveyed the store’s atmosphere through images of the store and social media links as well as products.

To create the VR store experience, the researchers developed a 360-degree-based VR video at a small local boutique with IRB approval. 360-degree-based VR videos were selected as methods as they are an easy way for any retailer to adopt. The videos were recorded in the store after the business hours, using Richo Theta V 360 camera. The recorded videos were edited using iMovie on Mac, into approximately eight one-minute-long videos showing eight sections: entrance, women’s wear, men’s wear, jewelry and accessories sections, and so on. Background music that simulates a store-like atmosphere was also created by the researchers. For the website version, the official store website was used with the owner’s approval.

VR participants were asked to visit VR facilities in the university library for around 20–25 min. They first received a short orientation relating to research agreement and safety. Thereafter, they watched the VR store videos with VR headsets that were played through the software, Steam. The mono layout and 360-degree format on Steam were selected to play the VR videos because these settings were the most suitable option. They watched eight one-minute videos showing different parts of the store (e.g., entrance, women’s, men’s and accessories sections) by clicking each section. The participants looked, walked, and moved around the store environments shown in the VR videos. While exploring the videos, background music was played to construct an atmosphere similar to the store. After finishing all eight videos, they completed an electronic Qualtrics questionnaire in the same facility, which took 10–15 min.

For the website experience, the participants were asked to explore the official website of the store and then completed the Qualtrics questionnaire. The survey first asked whether they actually visited the website. If they answered that they did not, participants were asked to stop the survey. On completion, they were also provided an opportunity for a drawing selection as compensation.

### Measurements

The survey questions were developed based on earlier studies. Arousal (e.g., not aroused—aroused) and pleasure (e.g., annoyed—pleased) were measured by a 7-point Likert scale of four semantic items each, adopted from Donavan and Rossiter (1982). Store attractiveness (e.g., unattractive—attractive) was measured by a 7-point Likert scale of three semantic scales adopted and modified from Baek et al., 2018; Orth & Wirtz 2014. Respondents narrated how much they felt each item during the store experiences when answering arousal, pleasure, and store attractiveness. Respondents indicated the degree to which they agreed with the statement, using a 7-point Likert scale (1 = “strongly disagree” to 7 = “strongly agree”). Store familiarity, the moderator, was measured by one item modified from Netemeyer et al. (2004) (i.e., The The XYZ is a store of apparel and fashion products I am familiar with). The respondents answered with either “Yes” or “No.”

The measurements are summarized in Table 3. Additionally, in order to evaluate VR respondents’ opinions about the VR experience, the participants responded to an open-end question: “How was this VR experience?”.

**Table 3:** Exploratory factor analysis and reliability

Scale items	Factor loading	Eigen value	Variance explained (%)	Cronbach’s alpha
*Arousal*				
		2.20	55.06	0.82
Not aroused—aroused	0.65			
Sleepy—wide awake	0.79			
Calm—excited	0.78			
Sluggish—Frenzied	0.73			
*Pleasure*				
		3.13	78.20	0.93
Depressed—contented	0.86			
Unhappy—happy	0.90			
Unsatisfied—satisfied	0.88			
Annoyed—pleased	0.90			
*Store attractiveness*				
		2.33	77.74	0.91
Unattractive—attractive	0.80			
Bad—good	0.90			
Unfavorable—favorable	0.94			

## Results

The data analyses were conducted using JMP 14.2. Exploratory factor analysis (EFA) on maximum likelihood with varimax rotation confirmed that all items were acceptable with factor loadings over 0.5 (Hair et al., 2010). Cronbach’s alpha for all variables was over 0.7, presenting a satisfactory level (Hair et al., 2010). Table 3 shows the results of EFA and reliability tests.

### Hypothesis testing

The study considered that previous VR experience can be an important factor impacting VR users’ arousal and pleasure. That is, whether the VR store experience is the consumers’ first use of VR or not was regarded as an influential determinant. Thus, this study tested whether VR- experienced users, who already had an experience using VR (coded as 1), and first-time users, who had never used VR (coded as 2), were differently aroused and pleased by the VR store experience in this experiment. The regression results confirmed that there are no differences in arousal ($\beta$=− 0.24, p = 0.32) and pleasure ($\beta$=0.17, p = 0.44) caused by previous VR experience. Hence, the study did not control the effect of previous VR experience for further hypothesis testing.

Hypotheses were tested using regression analyses by coding the website as 1 and VR as 2. The results are summarized in Table 4 and show that the VR store experience elicited greater arousal ($\beta$=0.72, p < 0.001) and pleasure ($\beta$=0.51, p < 0.001) than the website; thus, H1a and H1b were supported. H2 investigated the moderating effect of store familiarity between store experience and arousal (H2a) and pleasure (H2b). The moderating regression models (H2a and H2b) displayed in Table 4 were not significantly changed from the original regression models (H1a and H1b). Thus, it was concluded that there was no evidence of a moderating effect due to store familiarity, rejecting H2a (F = 1.47, $\beta$ =− 0.12p = 0.23) and H2b (F = 0.78, $\beta$=− 0.09, p = 0.38). Finally, consumers with greater arousal ($\beta$=0.49, p < 0.001) and pleasure ($\beta$=0.71, p < 0.001) were more likely to perceive the store as attractive, providing support for H3a and H3b.

**Table 4:** Hypotheses testing results

	DV	IV	β	SE	t	VIF
H1a	Arousal	Store experience	.72	0.16	24.62***	1.00
		R^2^ = .09, F-value = 21.30, p-value = .000				
H1b	Pleasure	Store experience	.51	0.15	3.37***	1.00
		R^2^ = .05, F-value = 11.33, p-value = .000				
H2a	Arousal (moderator)	Store experience	.72	0.16	4.58***	1.00
		Store familiarity	.19	0.05	0.27	1.01
		Store experience*Store familiarity	− .09	0.10	− 1.21	1.00
		R^2^ = .01, F-value = 7.59, p-value = .000, F change = 1.47, Sig. F change = .23				
H2b	Pleasure (moderator)	Store experience	.50	.15	3.27**	1.01
		Store familiarity	.06	.05	1.20	1.01
		Store experience*Store familiarity	− .09	.10	− .88	1.00
		R^2^ = .06, F-value = 4.50, p-value = .01, F change = .78, Sig. F change = .38				
H3a	Store attractiveness	Arousal	.49	.05	9.47***	1.00
		R^2^ = .30, F-value = 89.73, p-value = .000				
H3b	Store attractiveness	Pleasure	.71	.04	16.57***	1.00
		R^2^ = .57, F-value = 274.51, p-value = .000				

^*^p < .05, **p < .01, ***p < .001

### Sentiment analysis and text analytics of participants’ opinions about VR store experience

**Fig. 2:**
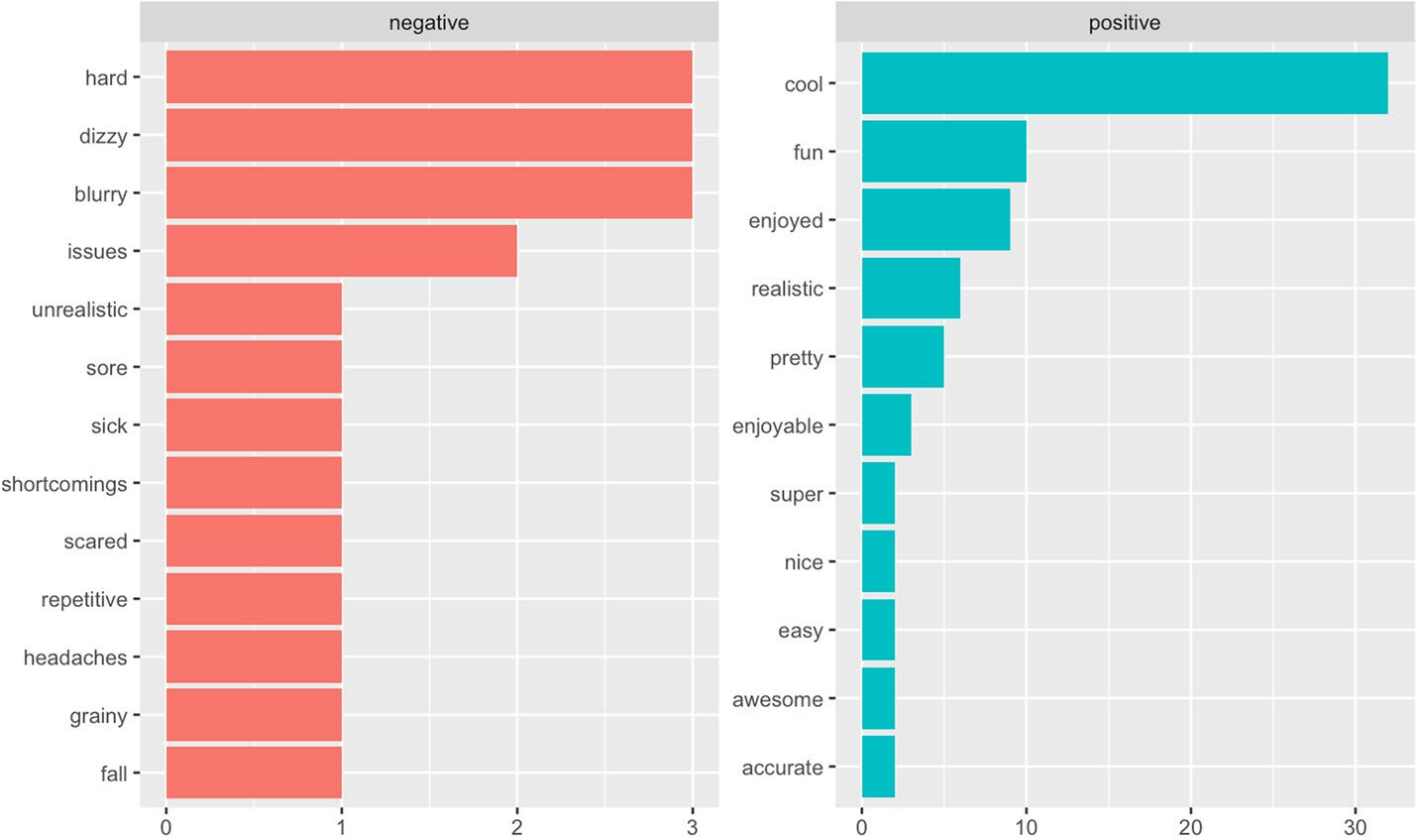
Sentiment analysis results: Commonly mentioned negative vs. positive words

The sentiment analysis and text analytics were conducted using the Tidytext package in the R studio software. First, the sentiment analysis was used to score the valence of opinions (i.e., positive or negative) using Bing Liu’s lexicon (Liu, 2012). The results revealed 82.30% positive and 17.70% negative words. Figure 2 shows the most common responses revealed by sentiment analysis. The left chart shows the negative word sets, while the right one presents positive words. Among the positive words, “cool” (n = 32), “fun” (n = 10), and “enjoyed” (n = 9) were ranked as the top three, followed by realistic, pretty, enjoyable. “Blurry,” “dizzy,”, and “hard” were mentioned three times each by participants, and ranked as the most commonly used negative words. Other negative words related to the physical discomfort were “sore,” “sick,” “headaches,” and “fall.”

**Fig. 3:**
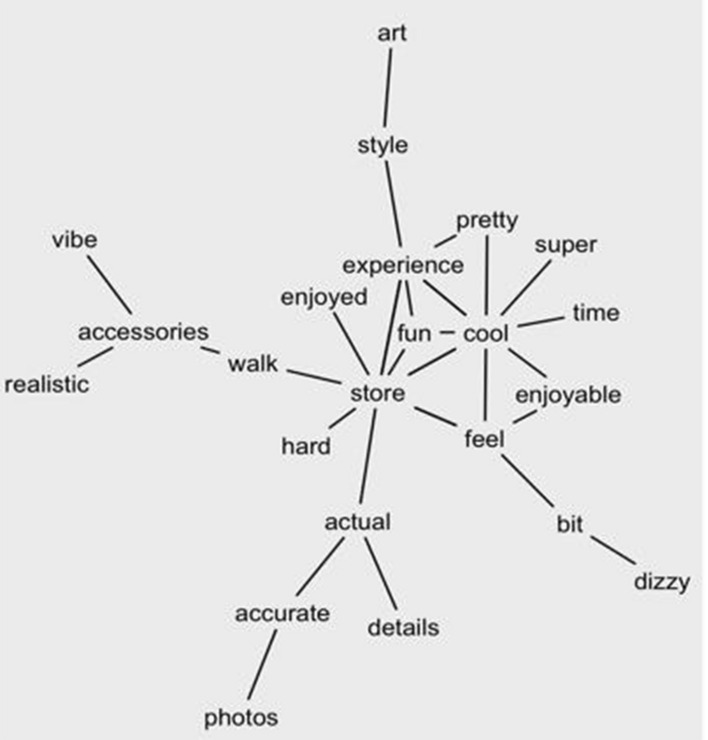
Semantic network analysis result using R: The relationship of two words reported commonly together

This study further conducted a semantic network analysis to understand how the mentioned words were related to each other. First, the top ten most frequently used words were selected: “store,” experience,” “cool,” “VR,” “feel,” “fun,” “enjoyed,” “time,” “music,” and “realistic”. Then, this study checked what two words were commonly reported together. This study identified several pairs of representative topic words: “experience; store,” “feel; store,” “cool; store,” “experience; cool,” actual; store,” and “enjoyed; experience.” There were also negative words: “bit; dizzy.” Fig. 3 presents how the words are related. There are 23 nodes and 28 relationships.

**Fig. 4:**
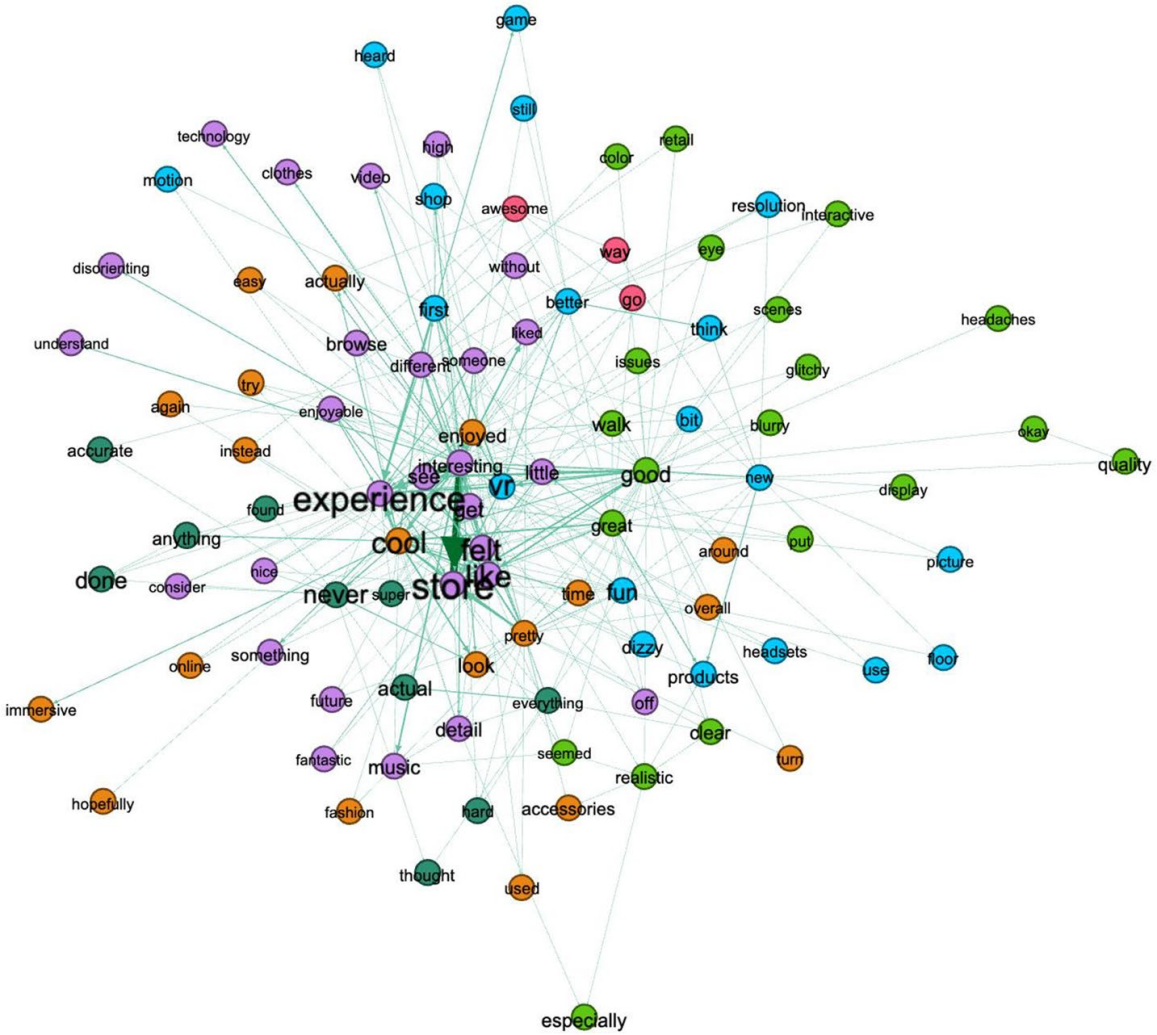
Semantic network analysis result using Gephi

To observe this more broadly, we further used Gephi 0.9.2. to draw a wider range of relationships based on representative words. Figure 4 shows the relationships among the entire word sets mentioned by the respondents after deleting words that were mentioned only once. In the figure, the nodes that are commonly mentioned are written in larger font sizes. The stronger (or weaker) relationships between any two words are presented as thicker (or thinner) lines.

## Conclusion and discussion

Retailers attract consumers through a unique shopping experience. One unique shopping experience consumers can enjoy online is through virtual reality (VR), as it can convey a store atmosphere without a physical visit. With this in mind, this study proposed a research framework based on the stimuli-organism-response model and the schema theory, tested with data collected in the U.S.

The results of this study indicated that the 360-degree VR store video can arouse (H1a) and please (H1b) consumers more than traditional websites can, supporting the literature. The finding of the study is in line with earlier research in the tourism field that found the effectiveness of VR, compared to 2D videos and static photos (Deng et al., 2019; Griffin et al., 2017). By overcoming the shortcoming of the website, VR amplifies consumers’ arousal and pleasure states. Furthermore, the findings are consistent with previous VR research in retailing, which confirmed that feelings present through VR positively influences pleasure and arousal (Park et al., 2018).

There was no support for a moderation effect of store familiarity between store experience (i.e., VR vs. website) and consumers’ emotional states (H2). The results conflicted with the literature on schema theory, because lower familiarity should be easier to change the perception towards a store than higher familiarity (Kent & Allen, 1994), presenting higher emotional states. The findings indicate that regardless of consumers’ store familiarity, VR is more effective in eliciting positive feelings compared to websites. This implies that the helpfulness of VR in evoking arousal and pleasure is not mitigated either in well-known or less-known companies. One possible explanation for this result is that consumers may have a weaker schema regarding VR than websites. Because the VR store experience remains novel to the consumers (Pizzi et al., 2019), it may elicit a greater change in their responses than the website. Therefore, the weaker schema related to VR may have a stronger impact on consumers’ responses than the weaker schema related to store familiarity itself. Accordingly, the findings offered the new empirical suggestion that store-related schema may have a weak explanation in the VR context.

Further, consumers’ heightened arousal and pleasure states were found to lead them to perceive a store as more attractive (H3). The outcome is in accordance with the previous literature that store-evoked arousal and pleasure augment positive consumer responses to the retailers (Eroglu et al., 2003; Ha & Lennon, 2010). Earlier studies also found that heightened arousal and pleasure through online store atmosphere and technology-related shopping experience elicited positive responses from consumers (Das & Varshneya, 2017; Dennis et al., 2014; Wu et al., 2008). This study further confirmed that store-elicited arousal and pleasure led consumers to find the store more attractive.

According to the findings of the sentiment analysis, consumers felt more positive emotion than negative toward the 360-degree-based VR-video experiences. The VR store experience was connected to positive words, such as “cool,” “feel,” “actual,”, and “enjoyed.” Furthermore, semantic network analyses discovered how the commonly mentioned words are related to each other. Several pairs, such as “experience; store,” “feel; store,” and “actual; store” were identified. These imply that consumers were able to experience and feel the store through VR videos. However, negative words were also identified by text analytics. These were related to VR quality, such as “blurry,” and user-related, such as “dizzy,” “sore,” “sick,” and “headaches.” Previous studies have discussed several negative emotions and side effects resulting from VR experiences, called VR sickness (Chang et al., 2020; Kim et al., 2018). The major symptoms of VR sickness include fatigue, nausea, headache, blurred vision, and dizziness. The study results denote that VR videos still have room for improvement to offer a better experience, supporting previous literature.

This study contributes to the literature in at least two respects. Academically, first, this study tested the VR store experience developed in an actual retail store. Previous studies pointed out the need for virtual environments other than 3D types (Gabisch, 2011) and of a virtual shop to portray a real-life retail store (Lau & Lee, 2018). While the replication of the actual place was used in tourism fields (e.g., Deng et al., 2019; Griffin et al., 2017), and a study in retail incorporated only static VR photos (Baek et al., 2020), this study extended the use of VR videos to the actual store context that was untested in previous literature.

Second, by using the store-recorded VR videos that can sufficiently deliver store atmosphere and environments, this study was able to link the VR store experience to consumers’ heightened emotional states and perception towards a store. While it is well accepted in the literature that VR store experience can lead to consumers’ behavioral intentions (Domina et al., 2012; Gabisch, 2011; Lau & Lee, 2018), the present study further focused on the perception towards a store atmosphere by emotions. The results added to the literature with regard to the role of customers’ VR store experience provoking emotions, applying the SOR model. Also, the exploration of an actual store through VR discovered that eliciting positive feelings led to heightened perceptions of store attractiveness, which has received little attention in the existing literature, compared to other responses. This discovery is unique, in that consumers feel the same store to be more attractive when experiencing the store through VR than websites. This study further added to the SOR literature in retail and marketing by incorporating a less tested variable—perceived store attractiveness—and by extending the model to compare the VR and website experiences.

Third, this study added to the literature by performing text analytics. Previous studies have mostly conducted experimental studies to reveal the effectiveness of VR and explain theoretically explained hypotheses. This study adds to this understanding by directly asking and exploring VR users’ opinions. In particular, the emotions evoked by consumers beyond arousal and pleasure were examined, illustrated by words such as “cool,” fun,” “enjoyed,” “blurry,” “scared”, and so forth as shown in Fig. 2. These semantic words provide the future direction of VR research observing various emotions other than arousal and pleasure driven by VR and leads to subsequent consumer responses.

On a practical side, first, with the discovery of the higher effectiveness of VR, compared to websites, this study suggests that retailers can utilize VR to create the perception of an attractive store environment, thereby bringing more consumers into the store (e.g., Baek et al., 2018; Darden et al., 1983; Orth & Wirtz, 2014). This presents an approach for online retailers to overcome physical limitations to offer store environments and atmosphere. Especially because the store attractiveness is led by heightened emotional states, arousal and pleasure, the VR store experience is suggested to be created in such a way as to enhance consumers’ and arousal and pleasure. In other words, retailers need to evoke consumers’ positive emotions through the shopping experience, to help consumers perceive the store attractive.

Second, this study found that the effectiveness of VR is consistent, regardless of consumers’ familiarity with the store. Therefore, any store, regardless of renown, can benefit from adopting VR technology.

Third, this research suggested a way to convey the store atmosphere to consumers without having them physically visiting the store, viz., recording VR store videos and uploading them to the website. The VR store experience used in this study consisted of 360-degree-based VR videos that recorded a physical store. Unlike the suggestion from earlier studies to create virtual stores graphically, this study recommends a simple method for any retailer. Retailers can record and post 360° store videos using 360° camera on their website or other video portals (Griffin et al., 2017; Lawton & Stapley, 2020), in addition to 2D store images. If the retailers upload VR video materials on their website, consumers can watch the videos with VR headsets from any location. Because simple 360° VR videos can encourage customers to perceive the store as attractive, and both the camera and VR headsets are inexpensive and widely available already, this method can be cost-effective for retailers, including small businesses.

Third, text analytics that analyzed an open-ended question about VR store experience offered more understanding to retailers. Based on the results of semantic network analyses, retailers are recommended to create more realistic but less dizzy VR environments and to decrease any possibility to cause discomfort. Some discomforts, conveyed through words such as “sore,” “blurry,” and “dizzy”, would need to be solved by the manufacturers of VR equipment. Thus, this study generates managerial implications and recommends that the VR manufacturers as well as retailers consider these discomforts.

## Limitations and future research

Despite the useful implications of this research, there are some limitations. First, the VR technology used in this study is the 360-degree-based VR video. This method is inexpensive for retailers but does not allow consumers to control the environment sufficiently. To create a more interactive atmosphere, other modes of VR usage can be included in further studies. Second, this study was conducted in a small boutique setting, where store familiarity was measured. For future studies, two stores varying by popularity can be used for experiments, such as comparing a large company (e.g., well-known globally) with a small company (e.g., its fame limited to a local area). Third, this study tested whether the VR store experience can help consumers find the store attractive. Further research is needed to explore whether this prediction of behavioral intentions through store attractiveness is consistent in the VR shopping context. For example, the relationship between store attractiveness displayed through VR and store visit intention can be tested. Fourth, we could not ensure that the same products were displayed in the VR videos and the website. The focus of the study was to compare the overall store experience delivered through the VR videos and website atmosphere rather than to examine the perceptions of individual items. Thus, future research may compare how the same items are perceived differently across these two stimuli.

## Data Availability

Not applicable.
